# Supplementary material for: Macrofluidic Coaxial Flow Platforms to Produce Tunable Magnetite Nanoparticles: A Study of the Effect of Reaction Conditions and Biomineralisation Protein Mms6
Source: Nanomaterials (Basel). 2019 Dec 4;9(12):1729. doi: 10.3390/nano9121729 (PMC6955933; doi:10.3390/nano9121729)
Supplement: Supplementary file 1 [file nanomaterials-09-01729-s001.pdf]

nanomaterials

Supplementary information

Macrofluidic Coaxial Flow Platforms to Produce Tunable Magnetite Nanoparticles: A Study of the Effect of Reaction Conditions and Biomineralisation Protein Mms6

Laura Norfolk [1,†], Andrea E Rawlings [1,2,†], Jonathan P Bramble [1,2,†], Katy Ward [1], Noel Francis [1], Rachel Waller [1], Ashley Bailey [2] and Sarah S. Staniland [1,2,*]

[1] Department of Chemistry, University of Sheffield, Brook Hill, Sheffield S3 7HF, UK; lnorfolk1@sheffield.ac.uk (L.N.); a.rawlings@sheffield.ac.uk (A.R.); jbramble82@gmail.com (J.B.); klward2@sheffield.ac.uk (K.W.); nfrancis1@sheffield.ac.uk (N.F.); rwaller2@sheffield.ac.uk (R.W.)
[2] School of Physics and Astronomy, University of Leeds, Leeds LS2 9JT, UK; bailey272@hotmail.co.uk
* Correspondence: s.s.staniland@sheffield.ac.uk; Tel.: +44-(0)114-222-9539
† These authors contributed equally to this work.

S1. Fluid dynamics modelling using COMSOL Multiphysics.

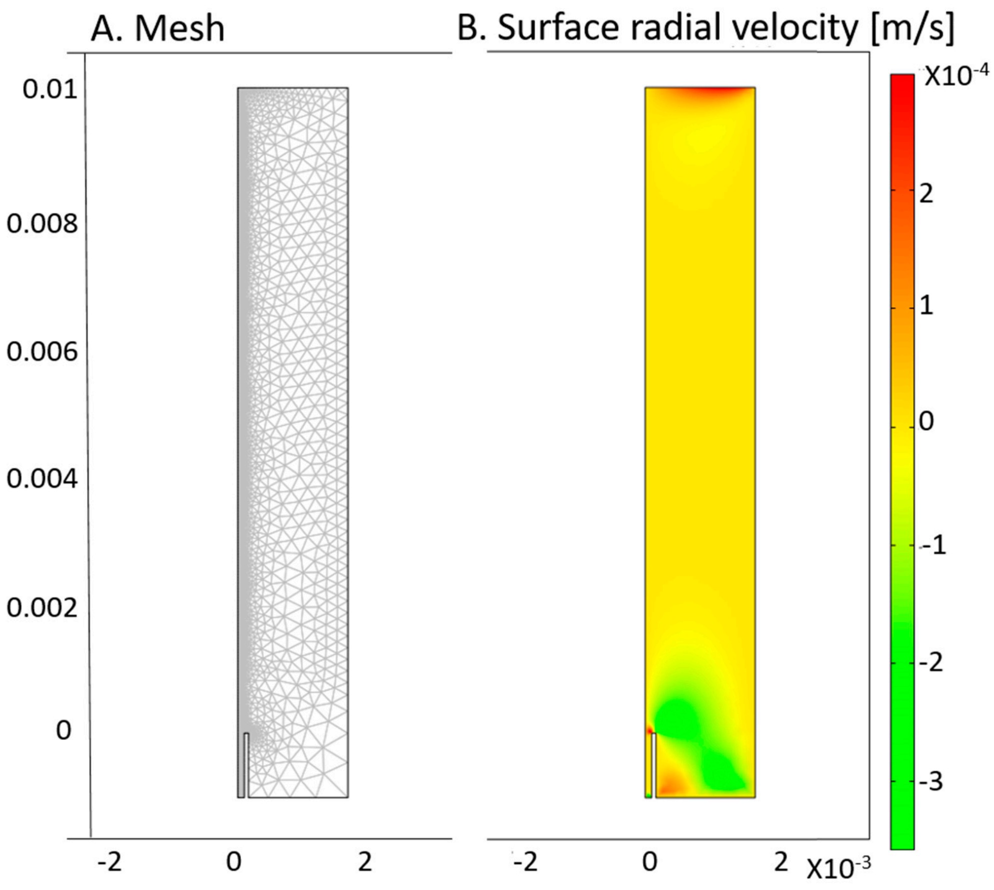

Figure S1. Demonstration of the fluidic dynamic modelled performed in COMSOL, x and y axis are length in metres (x is x 10^{-3}): A. shows the mesh used for the modelling. Note this is a radial symmetry model with the centre at 0, so only half the tube is shown. B. shows the surface radial velocity. The

yellow colour = 0 m/s showing negligible or no radial velocity indicating laminar flow (red at the end is an artifact of the boundary conditions).

S2. Comparison of both 1st generation PDMS and 2nd generation PEEK systems along with their resulting particles produced.

Table S1. Comparison of the specification of the two devices: tubing measurements and flow rates.

	Iron Solution (100 mM)		Sodium Hydroxide solution (IM)	
	Tube diameter	Flow rate	Tube diameter	Flow rate
1st generation PDMS device (Tube length 10 cm)	0.5 mm (0.02”)	60-70 μl min^{-1}	1.6 mm	300 μl min^{-1}
2nd generation PEEK device (Tube length 50 cm)	0.5 mm (0.02”)	60-90 μl min^{-1}	1.5 mm	240-360 μl min^{-1} (1:4 ratio)

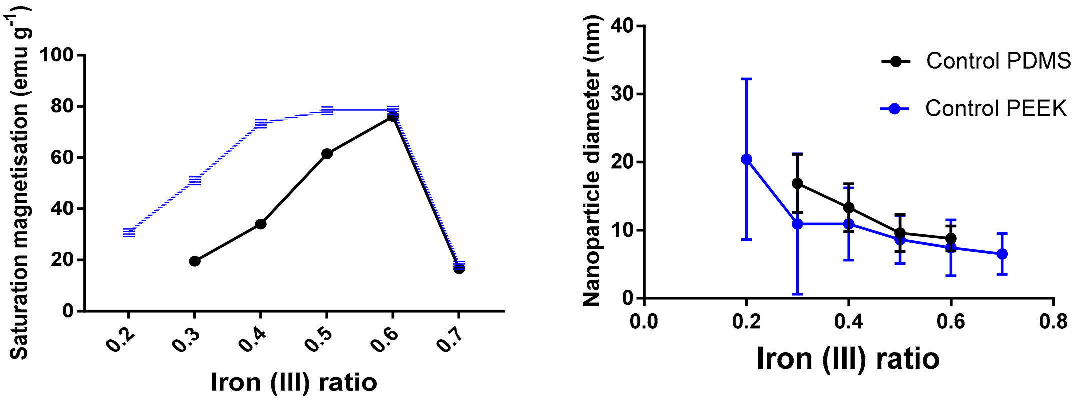

(a)

(b)

Figure S2. Variation of a) magnetic saturation and b) nanoparticle size verse the initial ferric to ferrous iron composition for both the first generation PDMS fluidic system and the second generation PEEK fluidic system. Ratio is of ferric iron to total ferric + ferrous iron precursor. a) obtained from sizing > 200 particle per sample using imageJ, b) was measured on a benchtop magnetometer for the PDMS device and on the VSM for the PEEK system.

S3. PDMS coaxial flow device modified with a Magnetic trap

A magnetic trap was introduced to the PDMS device to investigate whether MNP were formed inside the device or matured into MNP in the collection vessel. All other conditions remained unchanged. The nanoparticles which were initially magnetic and collected in the magnetic trap (primary products) and those collected in the final collection vial (secondary products) could then be compared with the total produce from the earlier unmodified study. It was clear to see visually that most of the magnetic sample was indeed trapped and was thus formed in the device and not subsequently.

There was a small amount of magnetic material in the secondary product, but it is difficult to say whether these particles were magnetic when leaving the device and failed to be trapped due to high flow rate or if they matured to become magnetic at a later stage. Nevertheless, the quantities are so minimal that we are confidence that the main magnetic iron oxide produce is produced w*ithin the co-axial flow device.*

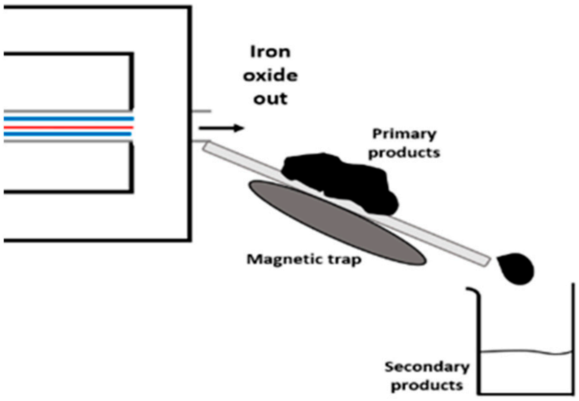

(**a**) (**b**)

Figure S3. a) Adapted outlet of PDMS co-axial flow device, incorporating a magnetic trap. **b)** Magnetic susceptibility of primary and secondary products measured on a benchtop magnetometer.

S4. Detailed analysis of particle sizes for the PEEK 2nd generation system.

Table S2. Mean particle sizes of the control and Mms6 mediated particles produced in the PEEK system.

Sample	Control Particle size TEM (nm)	Mms6 Particle size TEM (nm)
0.2	13.1 ± 9.6 (35.7 ± 19.4) Mean 20.5 ± 11.8	10.1 ± 9.0 (36.1 ± 11.4) Mean 15.5 ± 18.1
0.3	6.9 ± 4.9 (31.2 ± 10.2) Mean 10.9 ± 10.3	10.3 ± 8.4
0.4	10.9 ± 5.3	7.9 ± 2.5
0.5	8.6 ± 3.5	9.7 ± 3.6
0.6	7.4 ± 4.1	7.2 ± 2.5
0.7	6.5 ± 3.0	6.5 ± 3.0

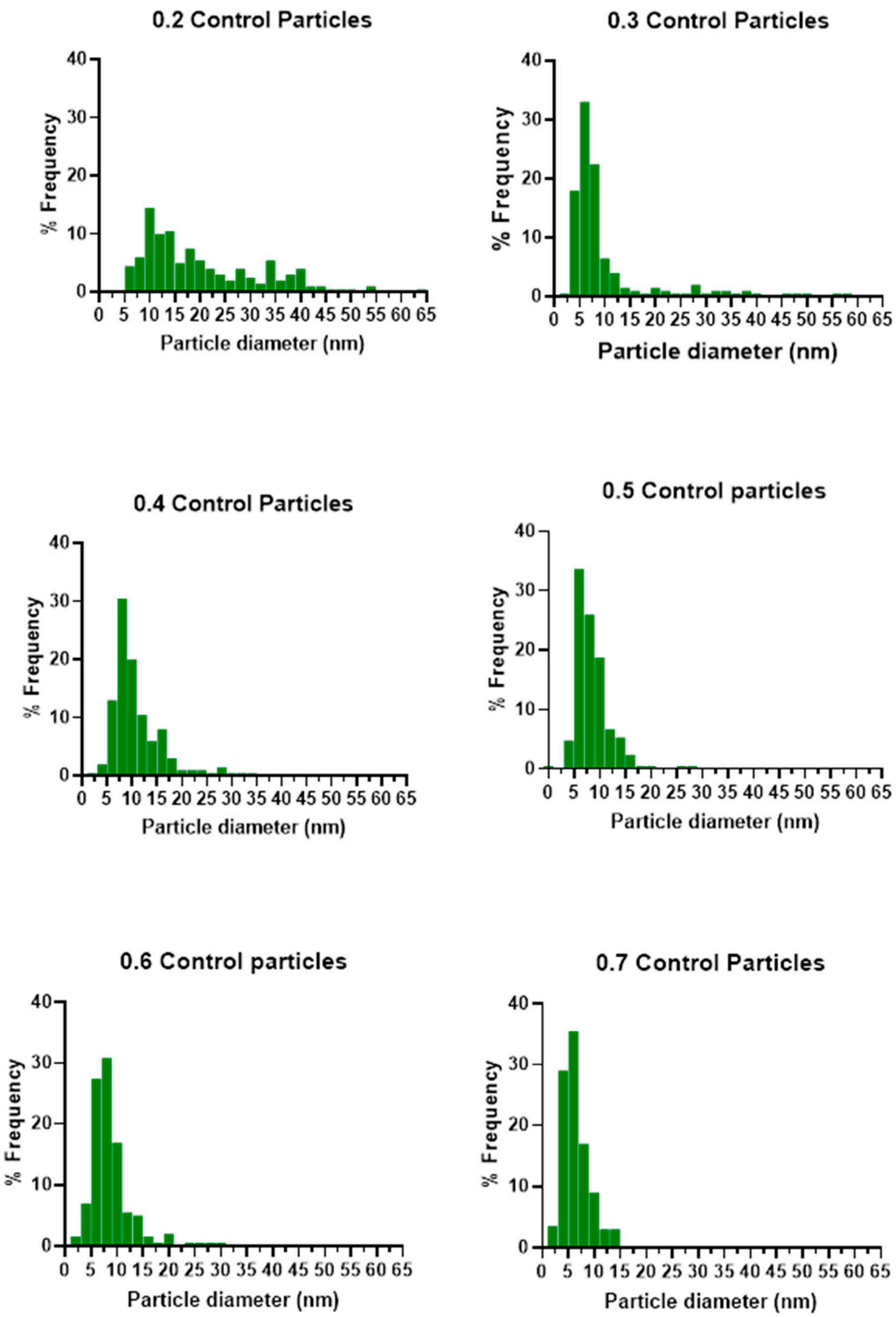

Figure S4a. Histogram series for the TEM size analysis of control samples.

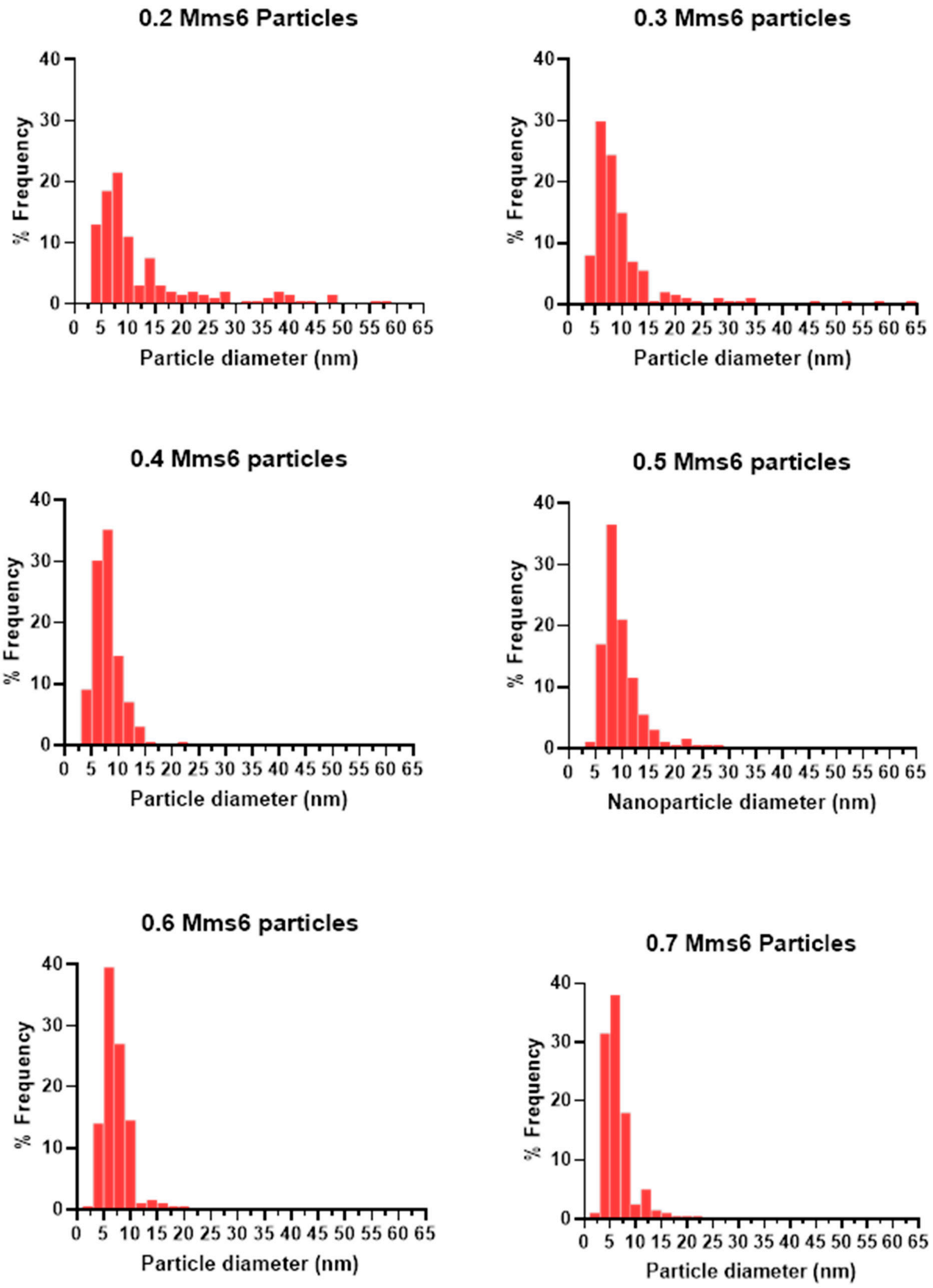

Figure S4b. Histogram series for the TEM size analysis of Mms6 mediated samples.

0.2 Control:

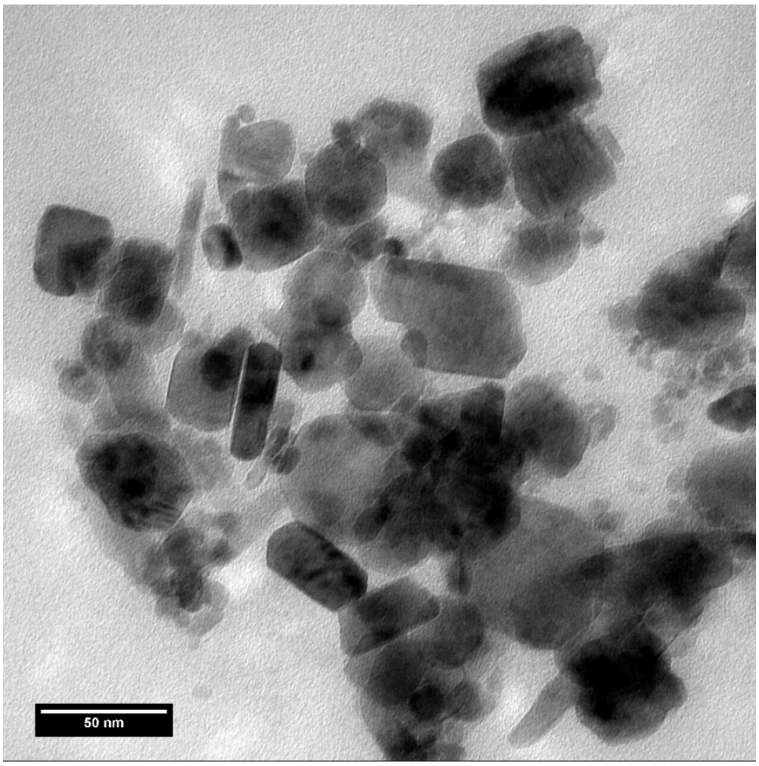

0.3 Control:

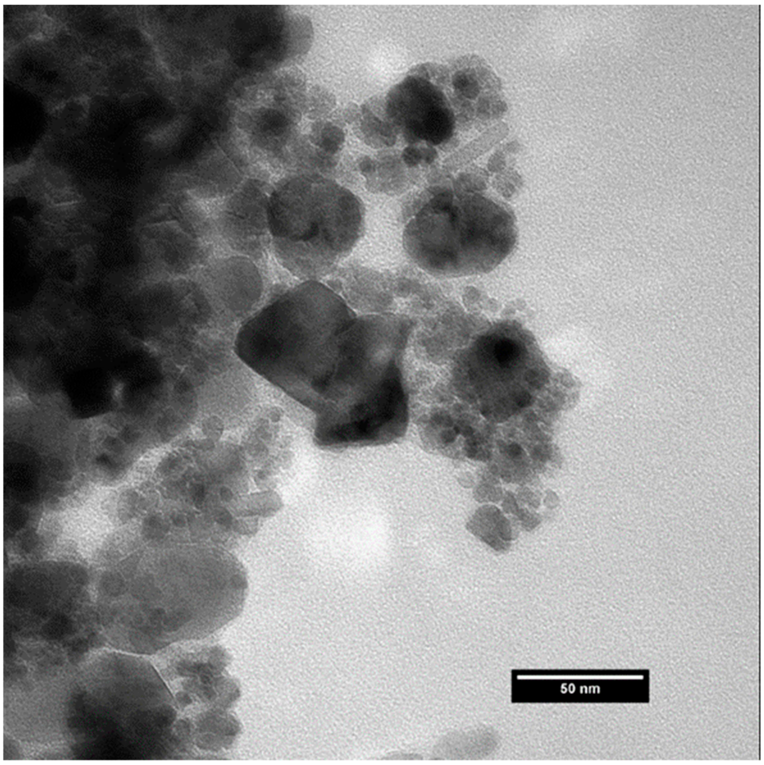

0.4 control:

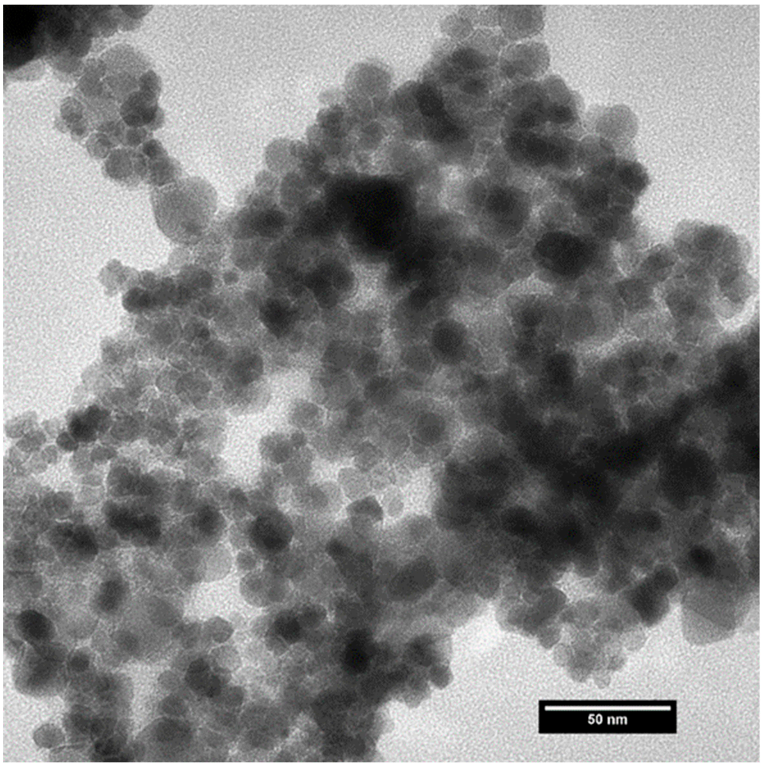

0.5 control:

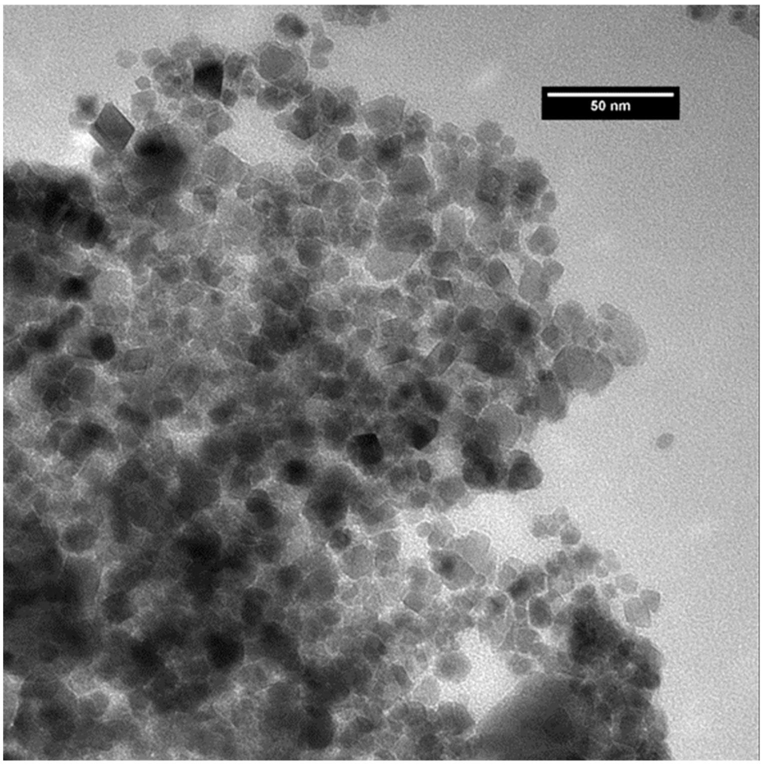

0.6 control:

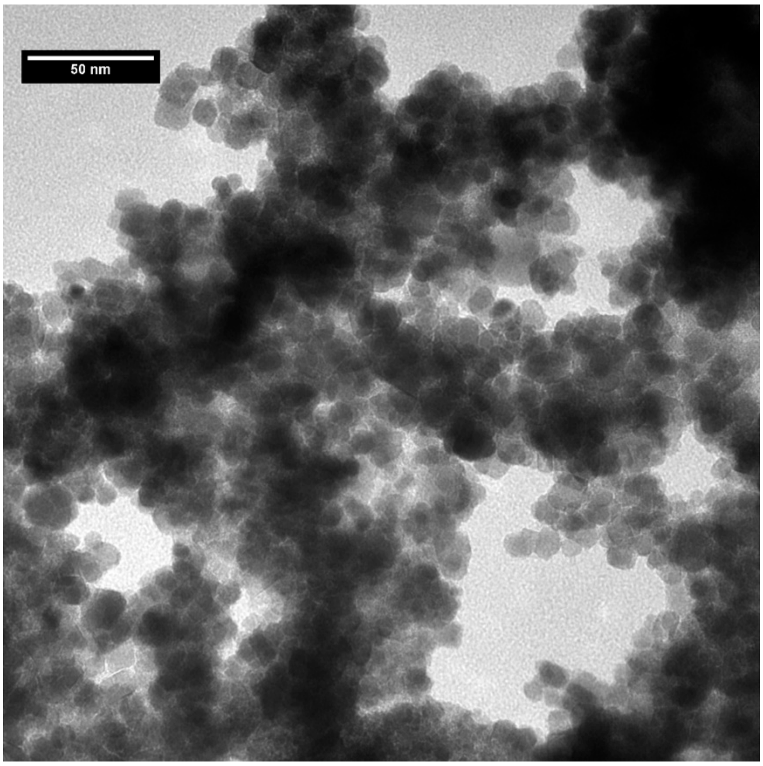

0.7 control:

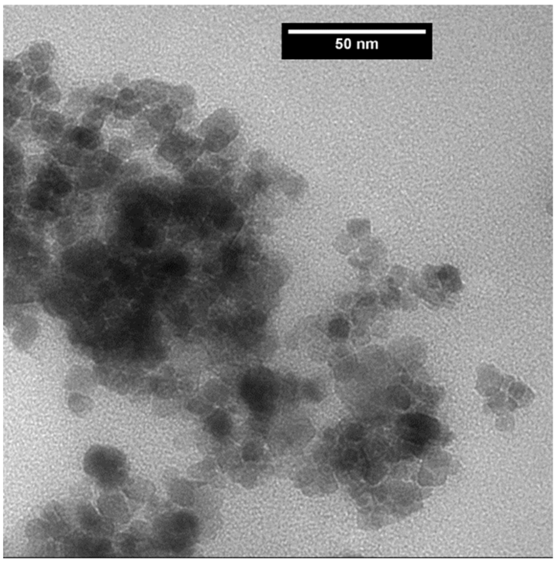

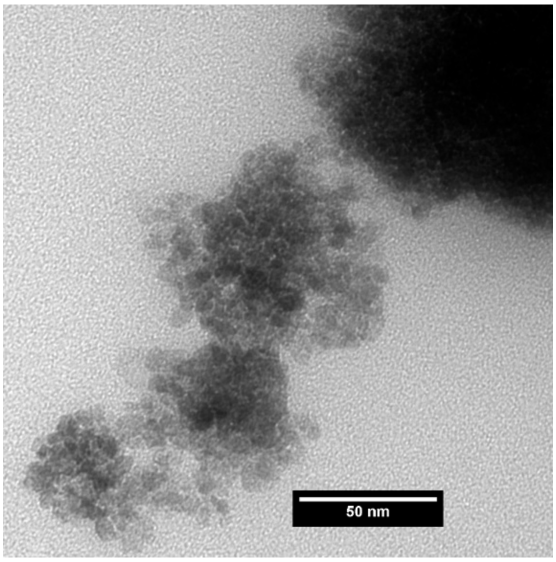

0.2 Mms6:

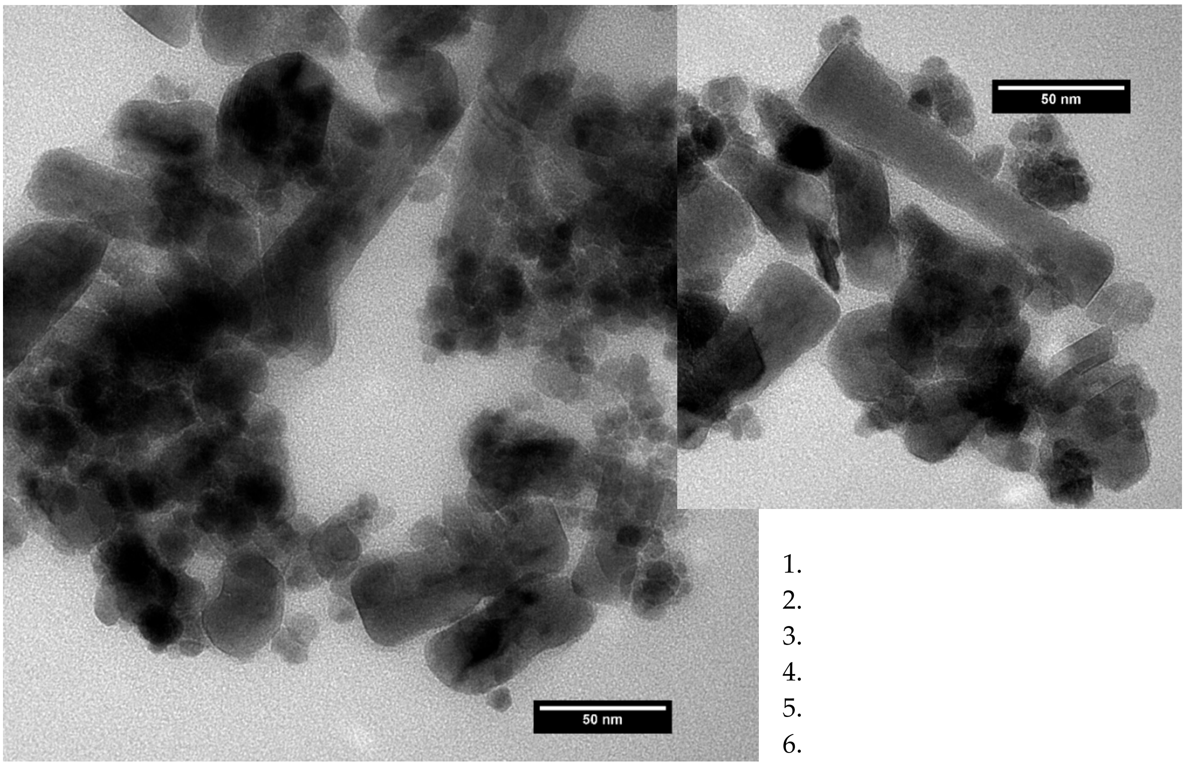

1.
2.
3.
4.
5.
6.
7.

0.3 Mms6:

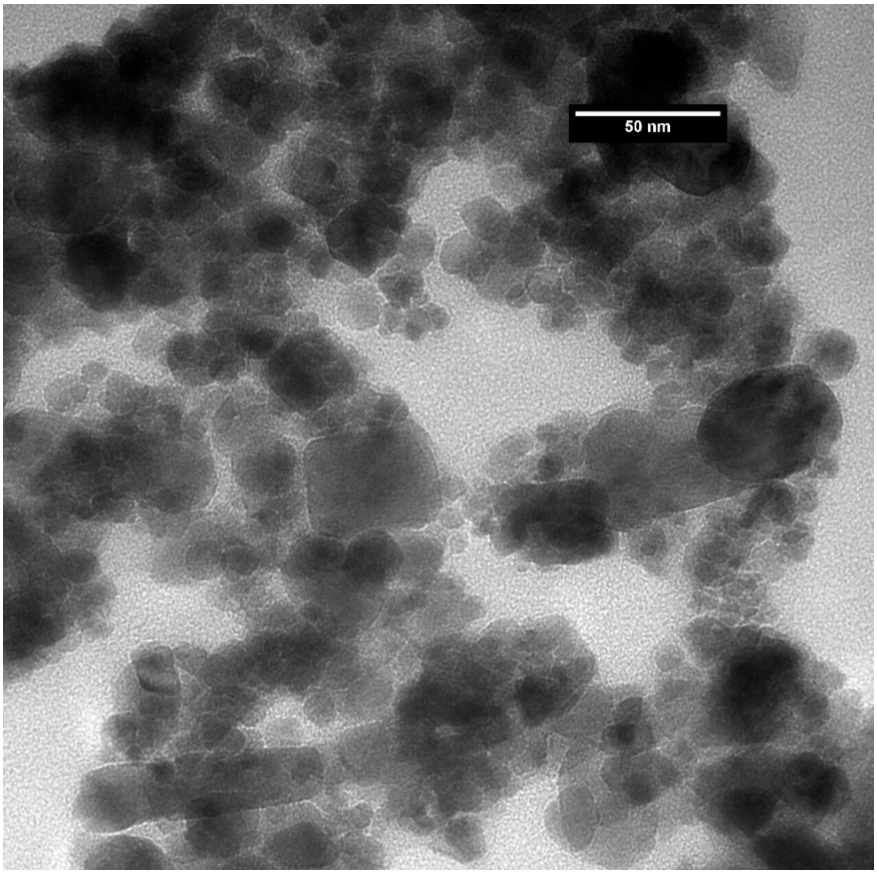

0.4 Mms6:

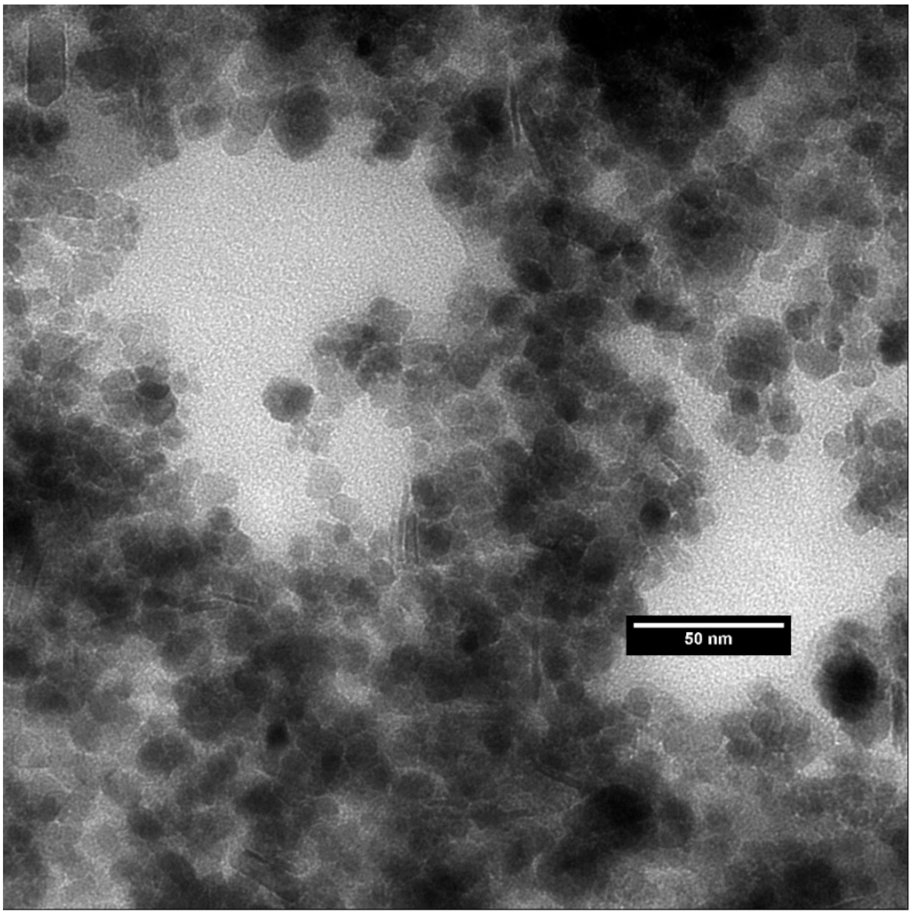

0.5 Mms6:

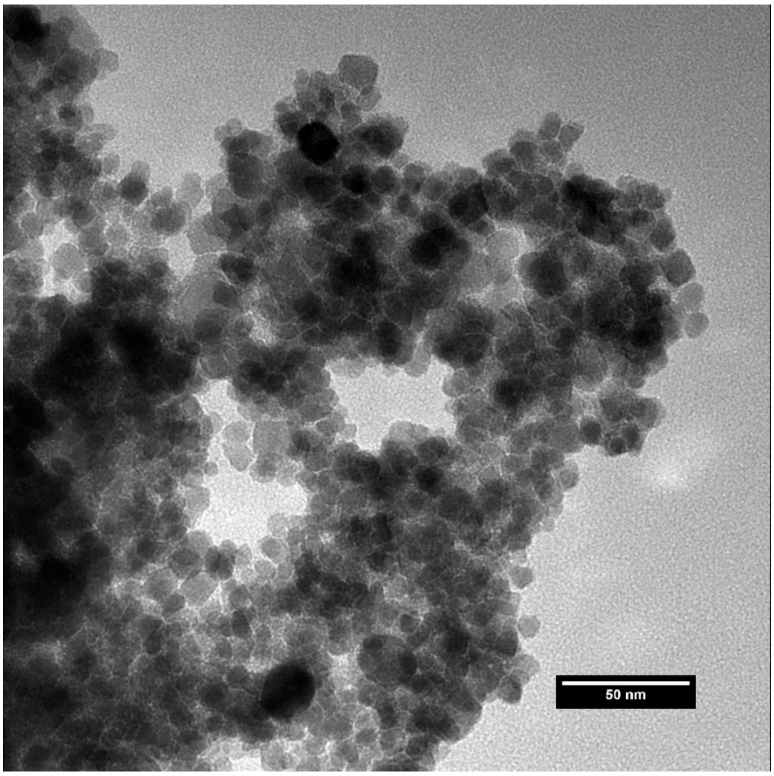

0.6 Mms6:

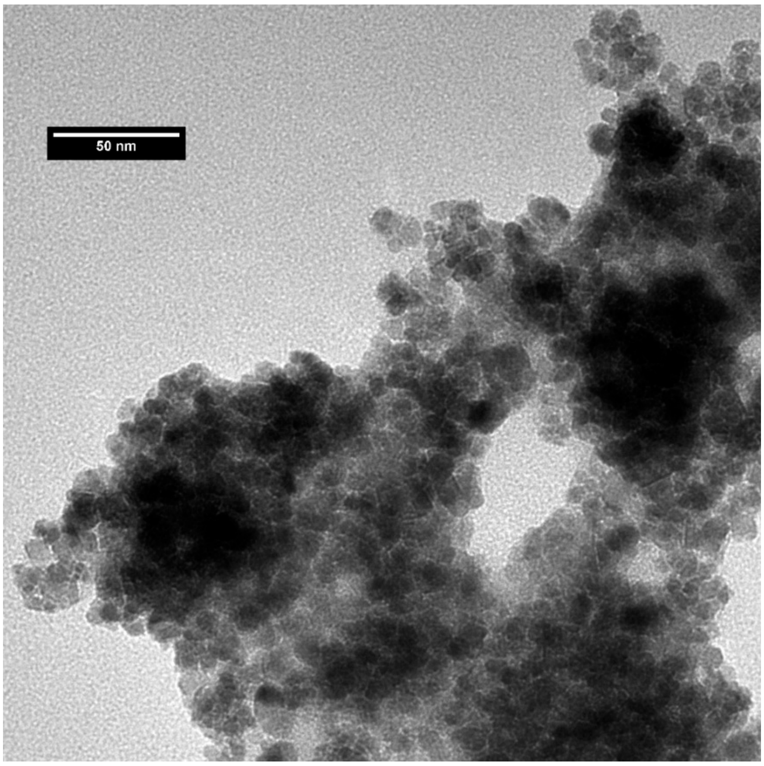

0.7 Mms6:

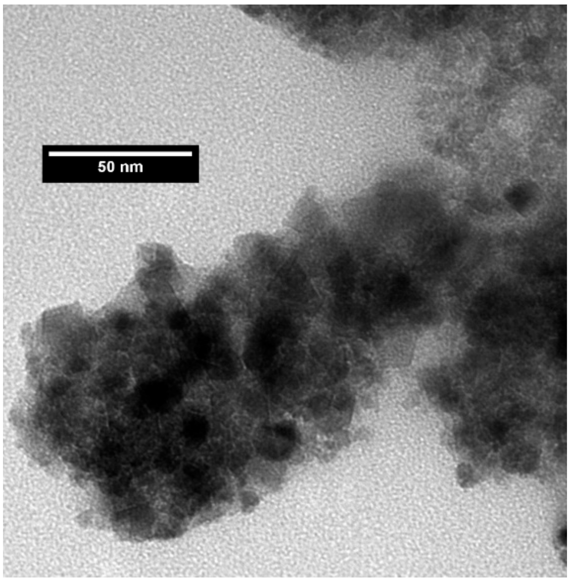

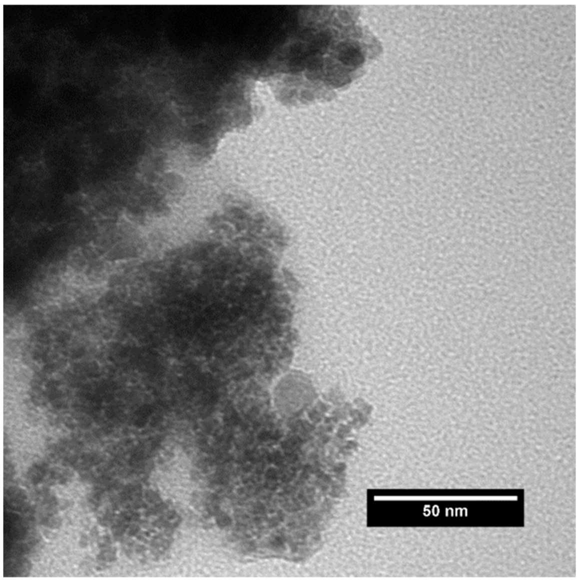

Figure S4c. Larger TEM Images of all samples.
